# SARS-CoV-2 and Acute Cerebrovascular Events: An Overview

**DOI:** 10.3390/jcm10153349

**Published:** 2021-07-29

**Authors:** Mehdi Ghasemi, Raffaella Pizzolato Umeton, Kiandokht Keyhanian, Babak Mohit, Nasrin Rahimian, Niloofarsadaat Eshaghhosseiny, Vahid Davoudi

**Affiliations:** 1 Department of Neurology, University of Massachusetts Medical School, Worcester, MA 01655, USA; raffaella.pizzolato@gmail.com (R.P.U.); kiandokht.keyhanian@gmail.com (K.K.); 2 Division of Pulmonary and Critical Care Medicine, Department of Medicine, Sleep Disorders Center, University of Maryland School of Medicine, Baltimore, MD 21201, USA; Bmohit1@alumni.jh.edu; 3 Department of Neurology, Tehran University of Medical Sciences, Tehran 1417613151, Iran; dr.nasrin.rahimian@gmail.com; 4 Division of Cardiovascular Medicine, Department of Medicine, Beth Israel Deaconess Medical Center, Harvard Medical School, Boston, MA 02215, USA; HOSSEININILOOFAR@yahoo.com; 5 Ann Romney Center for Neurologic Diseases, Department of Neurology, Brigham and Women’s Hospital, Harvard Medical School, Boston, MA 02115, USA; v_d904@yahoo.com

**Keywords:** coronavirus disease 2019 (COVID-19), severe acute respiratory syndrome coronavirus 2 (SARS-CoV-2), stroke, cerebral venous thrombosis, hypercoagulability, angiotensin-converting enzyme 2 (ACE-2)

## Abstract

Since the coronavirus disease 2019 (COVID-19) pandemic, due to severe acute respiratory syndrome coronavirus 2 (SARS-CoV-2) infection, accumulating evidence indicates that SARS-CoV-2 infection may be associated with various neurological manifestations, including acute cerebrovascular events (i.e., stroke and cerebral venous thrombosis). These events can occur prior to, during and even after the onset of COVID-19’s general symptoms. Although the mechanisms underlying the cerebrovascular complications in patients with COVID-19 are yet to be fully elucidated, the hypercoagulability state, inflammation and altered angiotensin-converting enzyme 2 (ACE-2) signaling in association with SARS-CoV-2 may play key roles. ACE-2 plays a critical role in preserving heart and brain homeostasis. In this review, we discuss the current state of knowledge of the possible mechanisms underlying the acute cerebrovascular events in patients with COVID-19, and we review the current epidemiological studies and case reports of neurovascular complications in association with SARS-CoV-2, as well as the relevant therapeutic approaches that have been considered worldwide. As the number of published COVID-19 cases with cerebrovascular events is growing, prospective studies would help gather more valuable insights into the pathophysiology of cerebrovascular events, effective therapies, and the factors predicting poor functional outcomes related to such events in COVID-19 patients.

## 1. Introduction

The coronavirus disease 2019 (COVID-19) pandemic due to severe acute respiratory syndrome coronavirus 2 (SARS-CoV-2) is the first one of the recorded pandemics that has caused a global burden on society and healthcare professionals. As of the date of writing this article, on 7 June 2021, over 173.41 million cases have been reported across 188 countries and territories, resulting in more than 3.73 million deaths, and over 2.13 billion people have been vaccinated [1]. The clinical manifestations of this disease are broad, ranging from asymptomatic cases to those with the severe symptomatic disease, with a case fatality rate of 2.3% [2]. The mortality is higher in elderly individuals, patients with medical comorbidities, and those with immunocompromised conditions [3]. While the primary mode of attack of the SARS-CoV-2 is through the respiratory pathways, early in the pandemic, reports from Wuhan, China, revealed that patients with COVID-19 might also develop neurologic symptoms (e.g., headache, dizziness and myalgia) [4]. Ever since, it has been found worldwide that neurological complications affecting both the central and peripheral nervous system (CNS and PNS, respectively) may occur in a considerable number of patients with COVID-19 [3,4,5,6]. The direct invasion of the nervous system by SARS-CoV-2 through the olfactory nerve, retrograde axonal transport, the gut–brain axis, or hematogenous spread has been suggested [6,7,8,9]. Critically ill COVID-19 patients admitted to the intensive care unit (ICU) may have additional risk factors for nervous system involvement, which include deep sedation and prolonged mechanical ventilation related to severe prolonged hypoxemia, immobility, and critical illness myopathy or neuropathy related to prolonged hospitalization, social isolation and delirium [10]. A correlation between SARS-CoV-2-related acute lung injury and brain hypoxia has been recently described, which may play an important role in the neurological dysfunction following SARS-CoV-2 infection [11,12].

Recent investigations have also indicated that some patients with COVID-19 may present with acute cerebrovascular events such as stroke [13,14] and cerebral venous thrombosis [15,16]. Although the mechanisms underlying such complications remain to be fully elucidated, the hypercoagulability state, hyper-inflammation, cytokine storm and cerebral endothelial dysfunction may play crucial roles [17,18,19,20]. In this review, we discuss the possible mechanisms underlying the acute cerebrovascular events related to SARS-CoV-2 infection, and also review the current epidemiological studies and case reports of neurovascular complications in patients with COVID-19 and relevant therapeutic approaches that have been considered worldwide.

## 2. Hypercoagulability Related to SARS-CoV-2

One of the important findings related to the SARS-CoV-2 infection is a widespread observation of the hypercoagulable state indicated by elevated D-dimer levels, the prolongation of the prothrombin time (PT), the activated partial thromboplastin time (aPTT), and abnormal platelet counts [18,21]. Both thrombocytopenia and elevated D-dimers can be justified by the disproportionate activation of the coagulation cascade and the use of its substrates; however, the pathophysiology of SARS-CoV-2-related coagulopathy is still debatable.

**Figure 1:**
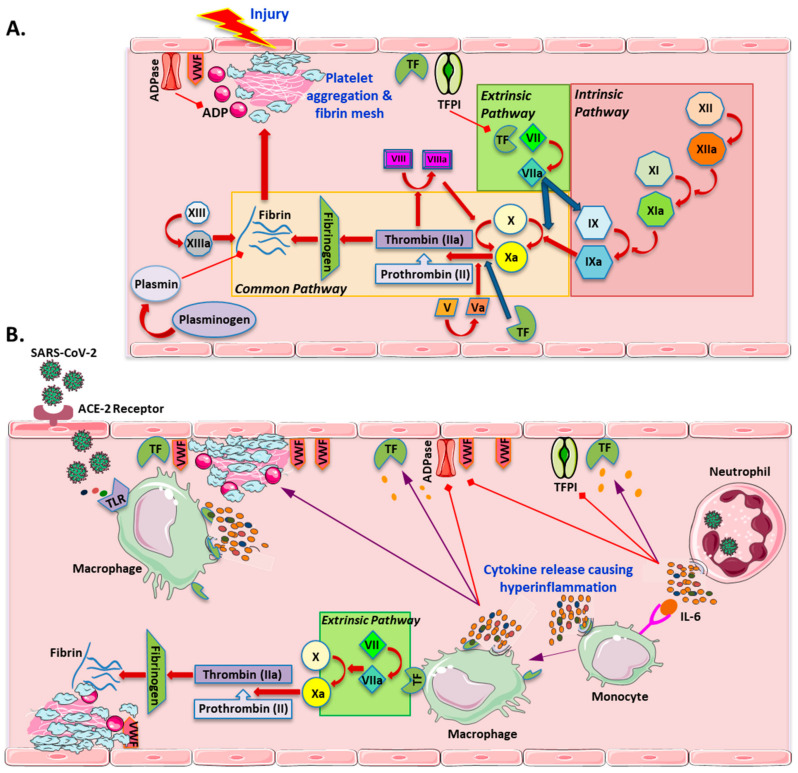
Schematic representation of (**A**) the normal coagulation cascade triggered by an injury to the endothelial cells and (**B**) the hypercoagulability state related to SARS-CoV-2 infection. Generally, with an injury to the endothelial cell, the first response is platelet aggregation, followed by the activation of both the extrinsic and intrinsic pathways that lead to the generation of factor Xa and common pathway initiation. The product of the secondary coagulation cascade is a fibrin clot which will enhance platelet aggregates. ADPase and tissue factor (TF) pathway inhibitors (TFPI), as well as plasminogen, act as regulatory anticoagulants. The extrinsic and common pathways are initiated due to the increased TF and von Willebrand factor (VWF) in response to inflammatory cytokines such as interleukin (IL)-6 and the expression of TF by mononuclear cells in response to IL-6, which is elevated in SARS-CoV-2 infection. TFPI and ADPase are inhibited by the cytokines as well.

While pneumonia itself can cause inflammation and a hypercoagulable state, cytokine release syndrome (CRS)- and macrophage activation-like syndrome (MAL)-like phenomena are also likely to play important roles [17,18,19,21]. When endothelial cells are damaged, generally, sub-endothelial cells—which are chromogenic—are exposed. The sub-endothelial cells contain Von Willebrand factor (VWF) and other thrombophilic proteins. Activated endothelial cells, along with VWF, will cause platelet aggregation and platelet plug formation as the primary homeostasis response (Figure 1). Secondarily, the coagulation cascade is activated, and it involves both extrinsic and intrinsic pathways, followed by a common pathway [22]. When the coagulopathy results from hyper inflammation and not an endothelial cell injury, the activated cascade would be an extrinsic pathway by the activation of a tissue factor or CD142 [23]. Tissue factor is expressed on mononuclear cells in response to interleukin (IL)-6 and other inflammatory cytokines, and will activate the extrinsic pathway [24]. Furthermore, inflammatory cytokines impose an inhibitory effect on anticoagulation regulators like tissue factor pathway inhibitors and ADPase [25,26]. Viral infection pro-coagulopathy seems to be both dependent on endothelial cells and innate immunity by the hyper-activation of toll-like receptors (TLRs) along the surface of monocytes, macrophages, dendritic cells and fibroblasts. Another possible contributor to this hypercoagulable phenomenon is the formation of acute reactive oxygen species and oxidized phospholipids due to acute lung injury, which seems to initiate the Toll-like receptor 4 (TLR4)−TRIF (TIR-domain-containing adapter-inducing interferon-β)−TRAF6 (TNF receptor-associated factor 6)−NF-κB (nuclear factor κ-light-chain-enhancer of activated B cells) pathway [27,28]. The downstream pathway for both the hyperactivation of TLRs in response to viral infection and acute lung injury involves NF-κB activation. As a result of the NF-κB activation, there will be more IL-6 and TNF-α, which are also bi-product of CRS and MAL reactions [19,29]. Coagulopathy was previously observed in infection with other Coronaviridae viruses, including SARS and Middle East respiratory syndrome (MERS) [27,30]. It has also been suggested that COVID-19 may induce antiphospholipid antibodies, but usually these antibodies are transient and found not to be pathogenic [31].

Although there are some prospective studies currently looking at the incidence of thrombotic events, early studies have already confirmed the increased frequency of intravascular thrombosis leading to pulmonary embolism, myocardial infarction, ischemic strokes and even cerebral venous sinus thrombosis. A thrombotic event has sometimes been reported as the first presentation of COVID-19 infection [18,32,33]. However, whether prophylactic anticoagulation for severe SARS-CoV-2 infection is beneficial still has to be considered according to common concurrent thrombocytopenia [17,21].

## 3. SARS-CoV-2 and Angiotensin-Converting Enzyme 2

**Figure 2:**
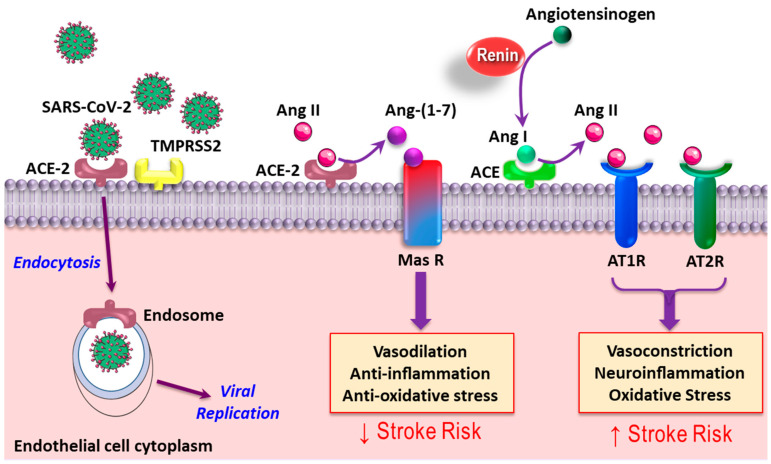
Schematic effects of SARS-CoV-2 on the renin–angiotensin system (RAS) in the cerebral vasculature. After the binding of the SARS-CoV-2 spike (s) glycoprotein to angiotensin converting enzyme 2 (ACE-2), the virus enters the cell. The virus also downregulates ACE-2 and competes with angiotensin II (Ang II) for binding to ACE-2, which ultimately decreases the activity of the ACE-2-Ang-(1-7)-Mas receptor (alternative) axis. This also leads to the greater activation of the ACE-Ang II-AT1R (classical) axis. The outcome of such events is an aberrant renin-angiotensin system (RAS), causing vasoconstriction, inflammation, oxidative stress, and thrombogenesis, causing ischemic stroke in relation to severe acute respiratory syndrome coronavirus 2 (SARS-CoV-2) infection. SARS-COV-2. TMPRSS2, Transmembrane protease, serine 2; AT1R: angiotensin 1 receptor; AT2R: angiotensin 2 receptor.

SARS-CoV-2 has spike (S) glycoproteins on its outer envelope, which have a strong affinity toward the human angiotensin-converting enzyme 2 (ACE-2) as the host cell receptor [34,35]. The binding of SARS-CoV-2 to ACE-2 is a crucial element for viral infectivity and multi-organ damage. ACE-2 is expressed in various human tissues such as CNS (glial cells and neurons), skeletal muscle, the gastrointestinal tract and endothelial cells [35]. In the cerebral vasculature, endothelial ACE-2, as part of the renin-angiotensin system (RAS), plays an important role in the modulation of cerebral blood flow. The key components of RAS are angiotensinogen, renin, angiotensin I (Ang I), angiotensin II (Ang II), ACE, ACE-2, Ang type-1 receptor (AT1R), Ang type-2 receptor (AT2R) and Mas receptor (Figure 2). Classically, the Ang II that is produced from Ang I by ACE activity mediates vasoconstriction, neuroinflammation and oxidative stress through the activation of AT1R and AT2R. Alternatively, Ang II can be converted to Ang-(1-7) by ACE-2 activity, which in turn activates the Mas receptor, mediating vasodilation, anti-inflammatory and antioxidant responses [36].

The overactivation of ACE/Ang II/AT1R/AT2R or the dysfunction of the ACE-2/Ang (1-7)-Mas receptor axis may contribute to the pathogenesis of acute ischemic stroke via increased vasoconstriction, oxidative stress and vascular inflammation (i.e., vasculitis) [36]. In SARS-CoV-2 infection, binding to ACE-2 may downregulate ACE-2 [37], leading to excess ACE-mediated Ang II production and lower ACE-2-mediated Ang-(1-7) production [20]. This SARS-CoV-2-induced imbalance between the classical and alternative RAS axes ultimately promotes ischemia via increased cerebral vasoconstriction, hyper-inflammation and oxidative stress [20]. Ang II promotes thrombosis by increasing the release and secretion of plasminogen activator inhibitor type 1 (PAI-1), and enhances tissue factor (TF) expression [38]. In contrast, the activation of the angiotensin-converting enzyme (ACE)2/angiotensin-(1-7)/Mas receptor would cause antithrombotic activity [39]. SARS-CoV-2 decreases the activation of ACE2; the result is an imbalance between the classical and alternative RAS axes, ultimately promoting ischemia via increased cerebral vasoconstriction, hyper-inflammation, oxidative stress and thrombogenesis [20]. These data raise the possibility that recombinant human ACE-2 might be beneficial in preventing ischemic stroke in COVID-19 patients with known stroke risk factors.

## 4. Acute Cerebrovascular Events in COVID-19

Prior articles indicate that viral respiratory infections are independent risk factors for both ischemic and hemorrhagic strokes [40]. Acute cerebrovascular events have been reported as one of the neurological complications that can occur in COVID-19 patients [41,42]; there is, overall, a propensity towards the occlusion of (i) large vessels (e.g., internal carotid, middle cerebral (M1 and M2 segments), or basilar arteries), (ii) multi-territory vessels, or (iii) uncommon vessels (e.g., pericallosal artery [43]) [44]. On the other hand, intracerebral hemorrhage, cerebral venous thrombosis and small-vessel brain disease develop less frequently in COVID-19 patients [44]. Several cases with atypical neurovascular presentations have also been reported, including bilateral carotid artery dissection [45], posterior reversible encephalopathy syndrome (PRES) [46], and vasculitis [47,48]. The pathophysiology, still unclear, is possibly related to the direct damage of the vessel mediated by the virus once it invades the CNS [49], or it could be related to the development of underlying coagulopathy and thromboembolisms, as we discussed earlier [33,50,51]. Another suggested mechanism is cardioembolic stroke from the direct damage of the myocardial cells, as evidenced by the cardiac dysfunction and arrhythmias in these patients [52]. Accordingly, myocardial cells express ACE-2 receptors abundantly, and are then vulnerable to SARS-CoV-2 infection [35].

In a retrospective study that included 214 patients with confirmed COVID-19 infection, about 6% presented with acute cerebrovascular events, mainly ischemic strokes. Stroke symptoms tend to appear later during the hospitalization, a median of 10 days after the onset of symptoms, and this was also confirmed by a larger retrospective study [53]. These patients seem to have a more severe infection with higher levels of inflammatory markers and higher D-dimer levels, older age, more comorbidities (hypertension in particular) and fewer typical symptoms associated with COVID-19 [54]. Indeed, for many COVID-19 patients presenting with acute strokes or other neurological manifestations, the diagnosis of infection is made after the hospital admission. The current recommendations from the American Heart Association (AHA) and American Stroke Association (ASA) include the use of personal protective equipment (PPE) for all of the stroke teams at the time of stroke code activation, as many stroke patients are unable to provide the history and information for appropriate COVID-19 screening. It is indeed suggested to treat every code stroke patient as being potentially affected by the infection in order to avoid any delay in trying to understand the infection status, following the same treating guidelines available for non-COVID-19 patients [55,56]. A dedicated track for the triage and management of suspected or proven COVID-19 patients with stroke-like symptoms was also suggested and implemented in Italy with a mobile CT scan unit [57]. The patients eligible for neurointerventional procedures should be treated accordingly, with the minimum number of staff in the angio suite and restricted access for essential staff, only ensuring the quality control of the negative pressure environment and following appropriate precaution protocols [58,59]. After the appropriate treatment, stroke patients should be admitted to a dedicated ward or ICU units where possible, and stroke teams should guide staff familiar with managing acute ischemic or hemorrhaging stroke patients [56].

### 4.1. Ischemic Stroke

Acute ischemic stroke appears to be the most common form of stroke seen in patients with COVID-19. The initial retrospective case reports from Wuhan in China reported six cases (2.34%) of stroke among the 214 patients analyzed, five of which were ischemic in nature [54]. Another study from Italy reported nine ischemic strokes (2.5%) among a cohort of 388 patients [51]. Different incidence rates were reported in two large studies. The first is a recent case series of 1419 patients with the diagnosis of COVID-19 admitted in a hospital in Madrid, Spain; it reported a total of 14 patients with systemic arterial thrombotic events (1% incident), of which eight presented with a cerebrovascular event (six with acute ischemic stroke and two with transient ischemic attack) [53]. A similar incidence (0.9%) was reported in the second large retrospective study of 3556 COVID-19 positive patients, of which 32 were diagnosed with ischemic stroke, 65.6% were defined as the cryptogenic subtype, and 34.4% were defined as an embolic stroke of undetermined source [60]. There is a possibility that the total numbers were underestimated because patients with small acute strokes may present without apparent focal neurological symptoms, and may go undiagnosed. Indeed, a case series from France documented three encephalopathic patients, with no signs suggestive of ischemic stroke, of whom the diagnosis was made after undergoing MRI to better address the cause of their encephalopathy [61]. Furthermore, the difference in the incidence rates could be explained by the different patient populations and larger cohorts. Another case series in Houston (TX, USA) reported a total of 12 patients with COVID-19 who developed stroke, among which 10 cases had an ischemic stroke (including one patient with hemorrhagic transformation), and two had intracerebral hemorrhage [62]. The inflammatory markers (e.g., D-dimer and IL-6) were elevated in a majority of these cases [62]. The etiology was an embolic stroke of undetermined source (ESUS, 6 cases), cardioembolic (2), carotid dissection (1), hypertension-related hemorrhage (1), the rupture of mycotic aneurysm related to infectious endocarditis (1), and unknown (one case due to limited workup) [62].

Stroke patients with COVID-19 infection usually present with a higher National Institutes of Health Stroke Scale (NIHSS) score at admission [60,63], a more severe disease course, immunocompromisation, and with different comorbidities and cardiovascular risk factors [53,54]. The age range is reported to be usually over 50 years old. However, more recently, a case series from New York City showed five COVID-19 patients younger than 50 affected by a large vessel ischemic stroke presented in the emergency department within a two-week period higher than usual (Table 1). Two of the five patients were previously healthy; one had hypertension and hyperlipidemia, another had undiagnosed diabetes, and the last reported patient had a history of prior mild stroke and diabetes [64]. The data from a larger patient cohort from New York City reported stroke in COVID-19 positive patients, mainly in men (71.9%) and white people (70%), with an average age of 62.5 versus 70 in the COVID-19−negative stroke patients. Moreover, patients with COVID-19 and ischemic stroke appeared to have higher mortality than the controls [60].

A large multicenter study reported stroke characteristics in 432 COVID-19 patients admitted to 71 centers from 17 countries. They observed a considerably higher rate of large vessel occlusions, a much lower rate of small vessel occlusion and lacunar infarction, and a considerable number of young strokes when compared with the population studies before the pandemic [63]. More data and studies on the incidence of stroke in young COVID-19 patients are needed.

A large international multicenter study on 17,799 COVID-19 hospitalized patients reported 156 stroke episodes, 123 (79%) of whom presented with acute ischemic stroke, 27 (17%) of whom had intracranial hemorrhage, and 6 (4%) of whom presented with cerebral venous sinus thrombosis. The mean age for ischemic stroke among hospitalized COVID-19 patients was 68.6 years [79]. Another multicenter prospective cohort study which included 150 patients with COVID-19 related ARDS showed 64 thrombotic complications, two of which were acute ischemic stroke despite anticoagulation [80].

Given the hypercoagulable state related to the infection, as a possible cause of ischemic stroke, prophylactic anticoagulation with low molecular weight heparin (LMWH) may be recommended for patients with severe COVID-19, according to the International Society of Thrombosis and Hemostasis (ISTH) [81]. The American Society of Hematology (ASH) guideline panel recently suggested: using prophylactic-intensity over intermediate-intensity or therapeutic-intensity anticoagulation for patients with coronavirus disease 2019 (COVID-19)–related critical illness who do not have suspected or confirmed venous thromboembolism (VTE) (conditional recommendation based on very low certainty in the evidence about effects). [82]

Higher mortality rates were observed in association with elevated PT and D-dimer levels, and decreased platelet counts and fibrinogen at days 10 and 14 from the onset of symptoms [81]. The monitoring of these parameters can help determine the prognosis and the selection of patients that require admission and aggressive treatments. Interestingly, in a retrospective study that included 449 patients with severe COVID-19 infection and elevated D-dimers, the use of LMWH was associated with lower mortality [83]. However, the data on the efficacy of LMWH in preventing venous and arterial thromboembolic complications are conflicting [51,80]. In a case report of six patients who presented with acute ischemic stroke and confirmed COVID-19 infection with associated elevated D-dimer levels (≥1000 μg/L), two patients had ischemic stroke despite therapeutic anticoagulation [13]. Data from the same study showed that the primary mechanism underlying the ischemic stroke was large-vessel occlusion, and the stroke usually occurred later in the course of the disease, between days 8 and 24 from the onset of symptoms. Further investigations are warranted to establish the actual need for therapeutic anticoagulation in patients with COVID-19 to reduce the risk of ischemic stroke.

PROTECT COVID (a randomized clinical trial of anticoagulation strategies in COVID-19) is ongoing, comparing the effectiveness of therapeutic versus prophylactic anticoagulation in patients with COVID-19 infection and mild-to-moderate elevations in D-dimer levels greater than 500 ng/mL (clinical trial identifier: NCT04359277) [60]. Other randomized trials are also ongoing to investigate the anticoagulation benefits in patients with COVID-19 (NCT04362085, NCT04345848, NCT04406389, NCT04528888).

Despite the lack of defined data on the prognosis of strokes related to COVID-19 infection, the overall outcome appears to be poor, as the majority of stroke patients are older and present with severe infection and more comorbidities [53,56,84,85,86]. Nonetheless, mechanical thrombectomy for emergent large vessel occlusion could be justified, as it can improve the outcome and should be offered to all the potential candidates notwithstanding the infectious status [59].

### 4.2. Hemorrhagic Stroke

A small number of stroke patients with COVID-19 infection present with cerebral hemorrhage (Table 1). The initial retrospective case series of 214 patients from Wuhan in China [54] reported only one case of hemorrhagic stroke. Similarly, another retrospective case series, again from Wuhan, reported one hemorrhagic stroke within 13 patients who presented with acute cerebrovascular events [86]. An additional five case reports of hemorrhagic stroke have been published [8,54,55,56]. A ruptured aneurysm in the pericallosal region [74] or posterior-inferior cerebellar artery (PICA) [14] was found in two cases. A hypothesis about the underlying pathophysiologic mechanism of cerebral hemorrhage is the reduced expression and function of ACE-2 in SARS-CoV-2 infected cells. ACE-2 is expressed in vascular endothelial cells, and its signaling is involved in the regulation of cerebral blood flow and the reduction of the body’s blood pressure. In the case of COVID-19 infection, the signaling is altered with subsequent hypertension and predisposition to the development of hemorrhagic stroke from arterial wall rupture [43]. Another possible mechanism is the underlying coagulopathy induced by the infection with thrombocytopenia [85]. Future observations may better clarify the incidence and clinical and laboratory characteristics of COVID-19 patients presenting with hemorrhagic strokes.

### 4.3. Cerebral Venous Thrombosis

Cerebral venous thrombosis has been reported in several studies. In a multinational retrospective study, all of the cases of cerebral venous sinus thrombosis (CVST) with COVID-19 infection were collected from the start of the pandemic to the end of June 2020. They reported on 13 post-COVID-19 CVST patients and compared their characteristics with the CVST data obtained before the COVID-19 pandemic from the same centers. They concluded that compared to non-COVID-19-infected CVST patients, patients with the infection tended to be older, and had fewer CVST risk factors and worse outcomes [87].

Several smaller studies reported CVST in seven adults (age range between 32 and 62 years, 62.5% female; Table 1) and one pediatric (a 13-year-old male) patient with COVID-19 [16,32,76,77,78]. Headaches were a presenting symptom in six (85.7%) cases, variably accompanied by different focal neurological deficits, confusion and impaired consciousness [16,32,76,77]. Although in three patients the treatment and outcome were not reported [16,77], the condition was fatal in three out of five cases (60%) within a few days of onset despite anticoagulation and supportive therapy. Notably, in some cases, neurological symptoms occurred about two weeks after the onset of the general symptoms (i.e., fever, cough or dyspnea) of COVID-19 [16,77]. Therefore, the possibility of this potentially life-threatening condition should not be overlooked even when patients present several days to weeks after the onset of COVID-19. A more recent multicenter 3-month cohort study of 13,500 consecutive patients with COVID-19 in New York City found an imaging-proved cerebral venous thrombosis incidence of 8.8 per 10,000 cases, which is higher than expected (i.e., 5 per million annually) [88]. In this study, despite the standard management [89] consisting of anticoagulation, endovascular thrombectomy and surgical hematoma evacuation, the mortality rate was 25% [88].

Overall, various degrees of elevated acute phase reactants (e.g., CRP and ferritin), hypercoagulability factors (e.g., D-dimer and aPTT) and abnormal platelet counts were found in these cases [16,32,76,77], suggesting a possible association with the hypercoagulability state in the setting of SARS-CoV-2 infection. It is unclear whether the monitoring of these markers has any value for the prediction of the onset or severity of cerebral venous thrombosis in these cases. This needs further detailed information on COVID-19 patients with such complications.

## 5. Therapeutic Approaches

Administering tissue plasminogen activator (tPA) in patients with COVID-19 and stroke is one of the therapeutic options. The role of other anticoagulants, such as low molecular weight heparin (LMWH) or full-dose heparin, is uncertain. There is some data to show that LMWH may be useful in sepsis-induced coagulopathy [83]. Although aspirin therapy in COVID-19 patients with ischemic stroke (especially in those who cannot take anticoagulants due to the risk of hemorrhagic transformation [67] or other medical limitations) can be considered as a secondary preventive approach, this medication is not indicated in patients with disseminated intravascular coagulation, a high risk of bleeding, or thrombocytopenia [90,91].

One reasonable treatment for COVID-19 patients is human recombinant soluble ACE-2 (hrsACE-2). There are two mechanisms of action for it: (1) preventing the SARS S protein from binding to lung and endothelial endogenous ACE-2, thereby reducing the infection of host cells; and (2) inhibiting the ACE-2 depletion by the SARS-CoV-2 virus. Considering ACE-2 is exhibited by brain endothelium and neurons, it is probable that the depletion of ACE-2 by the virus damages the endothelial function and leads to acute stroke. Along with the other known treatments, medications that affect the RAS system, such as angiotensin (1–7), may be appropriate therapies for COVID-19. Angiotensin’s role is currently under evaluation in clinical trials (NCT04332666). In addition, AT1 receptor blockers (ARBs), such as losartan, could be preclusive in stroke [92]. On the other hand, another study has shown that early intravenous thrombolysis and immediate mechanical thrombectomy had poor outcomes in patients with acute ischemic stroke due to large vessel occlusion with COVID-19 [93]. Overall, more well-designed, randomized, controlled trials are needed to provide an evidence-based approach for the prevention or treatment of acute cerebrovascular events in patients with COVID-19 [94].

## 6. Conclusions

A growing body of evidence indicates that acute cerebrovascular events, including both ischemic and hemorrhagic strokes and cerebral venous thrombosis, may occur in patients with COVID-19. The underlying mechanisms of such events are still not completely understood. Still, they may include a hypercoagulability state, inflammation and cytokine storm, endothelial dysfunction, and an aberrant RAS axis due to the binding of SARA-CoV-2 to endothelial ACE-2. These abnormalities ultimately cause vasoconstriction, oxidative stress, inflammation and thrombogenesis. As these complications, especially cerebral venous thrombosis, are potentially life-threatening, physicians need to be vigilant when encountering patients with COVID-19 who have neurological symptoms such as headache, confusion, altered mental status, seizure and focal neurological deficits. Due to the small number of published cases or the mainly retrospective design of the previous clinical studies, (i) the functional outcome with the available therapies (e.g., LMWH) for thrombotic events and (ii) inflammatory or coagulable markers that can be efficiently used for the monitoring or prediction of such events in COVID-19 are still elusive, requiring a large cohort of patients with such complications.

## Figures and Tables

**Table 1:** Case reports of acute cerebrovascular events related to COVID-19.

Region	Age, Gender	Neurological Symptoms on Admission (Day from Admission)	Other Symptoms (Onset Day Prior Neurologic Symptoms or after Admission)	Admission Serum Labs (or Day from Admission)	Imaging or EEG (Day from Admission)	Treatments Received	Outcome	Ref
Ischemic Stroke								
Wuhan, China	79, M	One-day right limb weakness, mild expressive aphasia (on exam)	Cough (7 days prior)	Lymphocytopenia, ↑ CRP (36.1 mg/L), ESR (43 mm/h), & lipoprotein(a) (1276 mg/L); normal cardiac, renal, &coagulation functions	Head CT scan: lacunar cerebral infarction. 48-h Holter monitoring: paroxysmal AF.	Oseltamivir, ribavirin, moxifloxacin, dexamethasone, clopidogrel, atorvastatin	Favorable; recovery within 12 days	[65]
London, UK	64, M	Mild left arm weakness, word-finding difficulty & incoordination (day 5); evolving to bilateral incoordination & right homonymous hemianopia (day 12)	Cough, dyspnea, fever, myalgia & poor appetite (10 days prior); evolving to respiratory failure (admission day) & PE (day 9)	↓ Hgb (119 g/L); ↑ LDH (654 U/L), ALT (137 U/L), PT (12.5 s), fibrinogen (950 mg/dL), D-dimer (>80,000 µg/L), ferritin (4927 µg/L) & CRP (305.4 mg/L); lupus anticoagulant (+); normal CBC_diff_, aPTT & INR.	Brain MRI (day 5): acute left vertebral artery thrombus and acute left PICA territory infarction with petechial hemorrhagic transformation. DWI MRI (day 12): bilateral acute PCA territory infarcts despite therapeutic anticoagulation. Lower limb Doppler ultrasound: occlusive DVT in left posterior tibial & peroneal veins. CT pulmonary angiogram (day 9): bilateral PE.	Initially aspirin & clopidogrel; then high-intensity LMWH anticoagulation (for PE)	Poor; ICU admission	[13]
London, UK	53, F	Acute confusion, incoordination, impaired consciousness (GCS 13/15)	Malaise, cough, fever & dyspnea (24 days prior)	↓ Hgb (94 g/L); leukocytosis (WBC 23K), ↑ LDH (664 U/L), PT (34.4 s), INR (3.6), aPTT (41 s), D-dimer (7750 µg/L), ferritin (1853 µg/L) & CRP (150.1 mg/L); lupus anticoagulant (+); Normal fibrinogen & LFT.	Head CT Scan: acute right parietal cortical & left cerebellar infarct with mass effect & hydrocephalus, despite therapeutic anticoagulation.	EVD for hydrocephalus & therapeutic LMWH anticoagulation	Death due to COVID-19 related cardiorespiratory failure	[13]
London, UK	85, M	Dysarthria, right facial droop, right-sided hemiparesis	Cough (10 days prior)	↓ Hgb (128 g/L); ↑ LDH (461 U/L), D-dimer (16,100 µg/L), fibrinogen (530 mg/dL), ferritin (1027 µg/L) & CRP (161.2 mg/L); lupus anticoagulant (−); normal CBC_diff_, PT, aPTT, INR & LFT.	Head CT Scan: hyperdensity consistent with thrombus in the left PCA & acute infarction in left temporal stem and cerebral peduncle.	Apixaban for AF	NR	[13]
London, UK	61, M	Acute dysarthria & left facial droop & hemiparesis (2 days prior COVID-19 symptoms started)	Fever, cough, dyspnea & tachypnea (2 days after admission)	↓ Hgb (126 g/L) & aPTT (24 s); thrombocytosis (408 K), ↑ LDH (444 U/L), fibrinogen (463 mg/dL), D-dimer (27,190 µg/L) & ferritin (1167 µg/L); lupus anticoagulant (+); normal CRP, PT, INR & LFT.	DWI brain MRI: acute infarction in the right corpus striatum suggesting transient occlusion of the M1 segment of the right MCA; FLAIR MRI: an established infarct in the same region with moderate background cerebral small vessel disease. CT pulmonary angiogram: pulmonary embolus in the left upper lobe segmental artery.	Therapeutic LMWH anticoagulation	NR	[13]
London, UK	83, M	Acute dysarthria, left facial droop & hemiparesis, & left-sided sensory inattention	Fever, cough, dyspnea & fatigue (15 days prior)	↓ Hgb (121 g/L); leukocytosis (WBC 11K), ↑ LDH (353 U/L), fibrinogen (496 mg/dL), D-dimer (19,450 µg/L) & CRP (27.7 mg/L); lupus anticoagulant (+); normal PT, aPTT, INR & LFT.	Head CT/CT angiogram: thrombotic occlusion of a proximal M2 branch of the right MCA; Repeat CT (24 h): a focus of parenchymal low density involving the right insular cortex in keeping with an evolving right MCA territory infarct	Intravenous thrombolysis	NR	[13]
London, UK	73, M	Acute aphasia & right facial droop & hemiparesis	Dyspnea & tachypnea (8 days prior)	Thrombocytosis (403 K), ↑ LDH (439 U/L), PT (12.3 s), D-dimer (1080 µg/L), ferritin (655 µg/L) & CRP (179.9 mg/L); lupus anticoagulant (+); normal PT, aPTT, INR & LFT.	DWI brain MRI: acute infarction in the right thalamus, left pons, right occipital lobe and right cerebellar hemisphere. Time-of-flight images: thrombotic material in the basilar artery and bilateral mild-to-moderate P2 segment stenosis.	Intravenous thrombolysis, decreasing D-dimer (1080 μg/L).	NR	[13]
New York, USA	33, F	28-h left hemiplegia, facial droop, gaze preference, homonymous hemianopia, dysarthria, sensory deficit, admission NIHSS 19	Cough, headache & chills (7 days prior)	↑ fibrinogen (501 mg/dL); normal WBC, platelets, PT, aPTT, D-dimer & ferritin.	Head CT/CT angiogram/MRI: partial infarction of the right MCA with a partially occlusive thrombus in the right carotid artery at the cervical bifurcation.	Apixaban (5 mg twice daily)	Favorable; complete resolution of thrombus in repeat CT angiogram (day 10), follow up NIHSS 13 (day 14)	[64]
New York, USA	37, M	16-h impaired consciousness, dysphasia, right hemiplegia, dysarthria, sensory deficit, admission NIHSS 13	No symptoms; exposed to family member with PCR-positive COVID-19	⭡ aPTT (42.7 s); normal WBC, platelets, PT, INR, fibrinogen, D-dimer & ferritin.	Head CT/CT angiogram/MRI: left MCA territory ischemic infarction	Clot retrieval, apixaban (5 mg twice daily)	Favorable; follow up NIHSS 5 (day 10)	[64]
New York, USA	39, M	8-h impaired consciousness, gaze preference to the right, left homonymous hemianopia, left hemiplegia, ataxia, admission NIHSS 16	No symptoms	↑ fibrinogen (739 mg/dL), D-dimer (2230 µg/L) & ferritin (1564 µg/L); normal CBC_diff_, PT & aPTT.	Head CT/CT angiogram/MRI: right PCA territory ischemic infarction	Clot retrieval, aspirin (81 mg/day)	Poor; multiple organ failure & intubated/sedated in ICU	[64]
New York, USA	44, M	2-h impaired consciousness, global dysphasia, right hemiplegia, gaze preference, admission NIHSS 23	Lethargy	↑ D-dimer (13,800 µg/L) & ferritin (987 µg/L); normal CBC_diff_, LFT, RFT, PT, aPTT & fibrinogen.	Head CT/CT angiogram/MRI: left MCA territory ischemic infarction	IV t-PA, clot retrieval, hemicraniectomy, & aspirin (81 mg/day)	NR; stay in stroke unit, follow up NIHSS 19 (day 12)	[64]
New York, USA	49, M	8-h impaired consciousness, left hemiplegia, dysarthria, facial weakness, admission NIHSS 13	Fever, cough & lethargy	↑ PT (15.2 s), aPTT (37 s), fibrinogen (531 mg/dL), D-dimer (1750 µg/L) & ferritin (596 µg/L); normal CBC_diff_, LFT & RFT.	Head CT/CT angiogram/MRI: right MCA territory ischemic infarction	Clot retrieval & stent, aspirin (325 mg/day), & clopidogrel (75 mg/day)	Favorable; follow up NIHSS 7 (day 4)	[64]
New York, USA	73, M	Acute altered mental status with hypoxemic respiratory failure (intubation)	Fever & dyspepsia (admission); nausea, vomiting & poor appetite (2 days prior)	⭡ CRP (26 mg/dL), prolonged PT (13.5 s), normal aPTT, LFT, & RFT; D-dimer, CRP & ferritin not checked.	Head CT scan: loss of gray-white differentiation at the left occipital & parietal lobes, consistent with acute infarction. Repeat CT head: progression toward a large acute infarct of the left MCA territory with hyperdense appearance of left MCA vessels - consistent with an acute thrombus.	Aspirin & supportive care	Poor; comfort measures only & terminally extubation	[66]
New York, USA	83, F	Admission: acute left facial droop, slurred speech, admission NIHSS 2. Day 3: left hemineglect, worsening left facial droop, & left hemiparesis, NIHSS 16	Fever & poor oral intake (admission)	Leukopenia & lymphocytopenia; D-dimer not checked.	Head CT/ CT angiogram (admission): No acute change, focal moderate stenosis of right MCA. Head CT (day 3): new moderate hypodensity in the right frontal lobe representing acute infarction.	Integrellin not started due to respiratory failure	Poor; respiratory failure & withdrawal of care	[66]
New York, USA	80, F	Acute altered mental status, aphasia & left side weakness, admission NIHSS 36	No symptoms; frequent falls (for 7 days)	Leukocytosis, lymphocytopenia, ⭡ D-dimer (13,966 µg/L), LDH (712 U/L) & CRP (16.24 mg/dL); prolonged PT (15.2 s); normal procalcitonin, aPTT, LFT & RFT.	Head CT scan: an acute right MCA stroke. Head/neck CTA: occlusion of the right internal carotid artery at origin. CT perfusion: a 305-cc core infarct in the right MCA distribution and a surrounding 109 cc ischemic penumbra.	Supportive care	Poor, comfort measures only and terminally extubation	[66]
New York, USA	88, F	Transient 15-min right arm weakness & numbness, & word-finding difficulty	Mild dyspnea & cough (admission)	⭡ D-dimer (3442 µg/L), CRP (12.7 mg/L) & IL-6 (8.5 pg/mL); prolonged PT (13.5 s); normal ferritin, procalcitonin, aPTT, Hgb, CBC_diff_, LDH, LFT & RFT.	Head CT scan: normal. Brain MRI: acute infarct in the left medial temporal lobe. Head/neck MR angiogram: mild stenosis of the right M1 segment	Aspirin & statins	Favorable	[66]
New York, USA	52, M	Acute global aphasia, right hemiparesis & left gaze preference, admission NIHSS 20	Fever, cough & dyspnea (7 days prior)	⭡ D-dimer (>10,000 µg/L), fibrinogen (235 mg/dL), ferritin (588 µg/L), CRP (11 mg/L) & ESR (37 mm/h).	Head CT scan: hyperdensity of the M1 segment of the left MCA. Head/neck CT angiogram: a left intracranial internal carotid artery occlusion. Repeat CT head: early infarct signs of in the left basal ganglia, internal capsule, caudate head, insular ribbon, operculum, & right posterior frontal lobe. CT perfusion: a favorable mismatch ratio of 4.1	IV alteplase, clot retrieval, aspirin, statin, & hydroxychloroquine	Partial recovery upon discharge	[67]
New Jersey, USA	84, M	Respiratory distress & unequal pupils	Fever, dyspnea, cough, & abdominal pain (14 days prior)	Lymphocytopenia, ↑ D-dimer (21,600 µg/L) & procalcitonin (0.25 ng/mL).	Head/neck CT/CTA: distal basilar artery occlusion extending into the proximal PCA, small aortic arch thrombus. Chest CT: bilateral lobar pulmonary emboli	LMWH (for PE); Clot retrieval	Death (Day 1)	[68]
Detroit, USA	72, F	Impaired consciousness, GCS 3 (day 10)	Progressive cough, myalgia, & dyspnea (21 days prior)	Admission: leukocytosis, acute kidney injury, transaminitis, & rhabdomyolysis; ↑ CRP & ferritin. Day 7: ↑ aPTT (28.5 s), PT (13.5 s), INR (1.32), mild thrombocytopenia (146K),	Head CT scan (day 10): bilateral cerebral infarcts in multiple vascular territories including cortical & subcortical regions.	Palliative care	Death	[69]
Philadelphia, USA	62, F	First admission: acute aphasia & right hemiparesis.2^nd^ admission (10 days after): altered mental status	No symptoms	Negative COVID-19 rRT-PCR in CSF (two times in 2nd admission).	Head CT angiogram (first admission): left MCA occlusion. Head CT scan (2nd admission): hemorrhagic conversion with midline shift & obstructive hydrocephalus	Clot retrieval (first admission), decompressive hemicraniectomy & EVD (2^nd^ admission)	Poor; stay in ICU	[14]
Los Angeles, USA	70s,	Acute aphasia, right hemiparesis & facial droop (day 5)	acute chest pain, diaphoresis, & hypotension with ST-elevation myocardial infarction (admission)	↑ aPTT (>85.5) while on heparin, renal failure, normal platelet count	Brain MRI: 60-cc acute infarct in the left insular, temporal, parietal, and frontal lobes, as well as smaller acute infarcts in the right caudate & left cerebellar hemisphere. MR angiogram: left MCA proximal M1 segment occlusion.	Palliative care	Poor	[70]
Toulouse, France	73, M	9-h acute aphasia & right hemiparesis, admission NIHSS 10	Fever & cough (7 days prior)	Lymphocytopenia, ⭡ CRP (219 mg/L), ferritin (109.6 µ/dL), fibrinogen (820 mg/dL) & D-dimer (2220 µg/L); normal platelets; negative antiphospholipid antibodies.	Head CT/CT angiogram/perfusion/MRI: subtle cortical left frontal hypoattenuation with more extended surrounding hypoperfusion & distal occlusion of left MCA branch; a large intraluminal floating thrombus appended to a hypoattenuated non-stenosing plaque of the left common carotid artery wall.	LMWH anticoagulation (enoxaparin)	Favorable; resolution of thrombus on carotid ultrasound (15 days after onset); follow up NIHSS 3 (day 10, discharge day)	[71]
Ronse, Belgium	74, F	Continued unconsciousness after extubation on day 16 & a mild endorotation of the arms	Fever, dyspnea & cough (7 days prior); evolving to hypoxic respiratory failure & intubation (admission day 3)	Slight lymphocytopenia, ⭡ CRP (18.79 mg/L), procalcitonin (1.93 ng/mL), ferritin (846.1 μg/L) & mild ⭡ creatinine (1.02 mg/dL); Day 2: ⭡ D-dimer (2504 µg/L). Day 16: ⭡ D-dimer (3941 µg/L) & fibrinogen (606 mg/dL); normal PT & platelets.	Head CT scan: large left MCA ischemic infarction with additional edema and midline shift; hyperdense artery sign was seen due to a thrombotic occlusion at the transition of the left internal carotid artery to the origin of the MCA.	Palliative care	Death (day 16)	[72]
Bilbao, Spain	36, F	48-h aphasia & acute right hemiplegia, admission NIHSS 21	No symptoms	Leukocytosis (23.6K), ⭡ CK (8669 U/L) & D-dimer (7540 µg/L) & CRP (15.6 mg/L).	Head CT scan: established infarct in the left MCA territory with a mild deviation of the midline. Head/neck CT angiogram: occlusion of the left internal carotid artery, MCA & ACA with a free-floating thrombus in the ascending aorta with no signs of aortic atheromatosis. CT pulmonary angiogram: bilateral PE.	Palliative care	Death within 72 h	[73]
Intracranial hemorrhage								
Philadelphia, USA	31, M	Acute headache & loss of consciousness	Malaise, fever, cough & arthralgia (7 days prior)	Negative COVID-19 rRT-PCR in CSF.	Head CT scan: SAH centered in the posterior fossa, including the 4th ventricle with hydrocephalus. Head CT angiogram: right-sided ruptured dissecting PICA aneurysm.	EVD for hydrocephalus & flow-diverting stent for aneurysm	Favorable; gradual improvement in mental status	[14]
Düsseldorf, Germany	60, F	Loss of consciousness	Concurrent respiratory insufficiency requiring intubation	Leukocytosis (14.2K), ⭡ troponin (45 ng/mL), CK (4920 U/L), CRP (11 mg/L), LDH (360 U/L) & GGT (103 U/L); normal CSF study.	Head CT/CT angiogram: left frontal haemorrhage with ventricle bleeding from a ruptured pericallosal artery aneurysm (~5 mm). CT perfusion (day 3, 6, 9 & 12): no cerebral vasospasm.	Aneurysm clipping & pneumonia treatment	Favorable	[74]
Brescia, Italy	57, M	Bilaterally fixed & dilated pupils, GCS 3 (day 11)	Fever & cough (7 days prior), worsening dyspnea (3 days prior)	Day 4: ↑ CRP, LDH, AST, & GGT.Day 11: prolonged aPTT (53.1 s), ↑ D-dimer (2866 µg/L).	Head CT scan (day 11): bilateral cerebellar hemispheric hemorrhage with 4th ventricle & brainstem compression and supratentorial hydrocephalus & diffuse obliteration of sulci. CT angiogram: Normal.	NR	Death (1 h after CT)	[43]
Brescia, Italy	57, M	bilaterally fixed and dilated pupils, GCS 3 (day 12)	Fever & cough (10 days prior), dyspnea (3 days prior)	Admission: ↑ CRP (21 mg/L), LDH (771 U/L), AST (100 U/L), GGT (152 U/L).	Head CT scan: diffuse cerebral edema with a large right frontal hemorrhage extending to ventricles.	LMWH (for PE) prior neurological deterioration	Death (shortly after CT)	[43]
Sari, Iran	79, M	Acute loss of consciousness	Fever & cough (3 days prior)	Lymphocytopenia, thrombocytosis (210 K); prolonged PT (12 s), INR (1), & aPTT (64 s); ↑ ESR (85 mm/h), CRP (10 mg/L), & creatinine (1.4 mg/dL); normal LFT	Head CT scan: massive right-hemispheric hemorrhage extending to ventricles & SAH	NR	NR	[75]
Cerebral venous thrombosis								
Wales, UK	59, M	First admission: 4-day progressive headache Second admission (4 days later): acute right sided weakness & numbness, slurred speech, expressive aphasia, admission NIHSS 10	First admission: fever & hypertension.	Prolonged aPTT (22.3 s), ⭡ CRP (15 mg/L) & creatinine (57 mg/dL); normal CBC_diff_, PT & fibrinogen.	Head CT scan (1st admission): hyperdensity within the superior sagittal sinus, right transverse sinus, sigmoid sinus & upper right internal jugular vein suggestive of venous thrombosis. Head CT venogram (1st admission): normal; however, it was re-reviewed 4 days later (2nd admission): filling defect in the right sigmoid & transverse sinus involving the torcula.	LMWH	Favorable; NIHSS 4 within 24 h	[32]
New York, USA	38, M	Seven-day headache & 2-day impaired consciousness; evolving to extensor posturing of the arms & clonus with NIHSS 14	Persistent diarrhea & vomiting (10 days prior)	NR	Head CT scan: Hyperdensity in the straight sinus, distal superior sagittal sinus, torcular & right transverse sinus, & in several cortical veins adjacent to the superior sagittal sinus, suggestive of cerebral venous thrombosis.Head CT venogram: near-occlusive thrombus in the right internal cerebral vein.	Enoxaparin (70 mg subcutaneously twice a day) & clot retrieval, lopinavir, ritonavir	Death within 32 h	[76]
New York, USA	41, F	Acute confusion, global aphasia & left gaze preference, admission NIHSS 16; rapidly worsening mental status & extensor posturing to noxious stimulation requiring intubation	Prior admission for COVID-19	⭡ D-dimer (2032 µg/L). CSF: ⭡ protein (616 mg/dL) & 41 WBC (PMN 84%), normal glucose.	Head CT/CT angiogram (admission): normal. Repeat CT head: a venous infarction in the left basal ganglia, thalamus, & mesial temporal lobe with hemorrhagic transformation, intraventricular hemorrhage, & obstructive hydrocephalus. Head CT venogram: occlusion of the internal cerebral veins.	EVD & heparin infusion	Death within 4 days	[76]
New York, USA	32, M	One-week headache & impaired consciousness	Concurrent 7-day fever & dry cough	↑ Glucose (1384 mg/dL), D-dimer (>11000 µg/L) & ferritin (18,431 µg/L)	Head CT scan: patchy areas of low density in the bilateral cerebral hemispheres with foci of subcortical hemorrhage in the left parieto-occipital region. Head CT angiogram: normal. Brain MRI: confluent, nonenhancing regions of pathologically reduced diffusion throughout the subcortical & deep hemispheric white matter bilaterally, left greater than right. Punctate foci of susceptibility artifacts consistent with petechial hemorrhage on gradient recalled-echo images, suspecting diabetic ketoacidosis related venous thrombosis.	Azithromycin, hydroxychloroquine	Death within few days	[76]
Rome, Italy	44, F	Ingravescent dyspnea, headache, impaired consciousness, aphasia & right hemiparesis	Fever, cough & dyspnea (14 days prior)	Neutrophilic leukocytosis, lymphocytopenia, thrombocytopenia (42 K); ↑ D-dimer (5975 µg/L), CK-MB (6.9 µg/L),; Negative anti-cardiolipin, -β_2_-glycoprotein & -dsDNA antibodies	Head CT angiogram: Dural sinus thrombosis with poor representation of left internal cerebral vein. Pulmonary CT angiogram: Filling defect within the inferior trunk of the right pulmonary artery and along the superior vena cava by thrombi.	NR	NR	[77]
Paris, France	62, F	Headache & altered vision, evolving to sudden right hemicorporeal deficit & impaired consciousness	Fever, cough &dyspnea (15 days prior)	Leukocytosis (20 K); ↑ AST (54 U/L), ALT (68 U/L), GGT (87 U/L), & D-dimer (14,200 µg/L)	Head CT scan & brain MRI: large confluent intraparenchymal hemorrhage in the left fronto-temporal lobes. CT venogram: cerebral venous thrombosis of the left transverse sinus, straight vein, vein of Galen and internal cerebral veins.	NR	NR	[16]
Paris, France	54, F	Severe headache	Fever & asthenia (14 days prior)	Leukocytosis (18K), ↑ CRP (170.8 mg/L) & D-dimer (2360 µg/L)	Head CT scan & brain MRI: large hemorrhagic infarction in the left temporal lobe. CT venogram and MR angiography: cerebral venous thrombosis of the left transverse sinus	NR	NR	[16]
Madrid, Spain	13, F	Impaired consciousness & intense headache	Fever, cough, & odynophagia (7 days prior) followed by frontal headache & vomiting	Leukocytosis (14.4 K), lymphocytopenia, thrombocytopenia; ↑ D-dimer (33,960 µg/L), CRP (12.55 mg/dL), LDH (322 U/L), & ferritin (240 ng/mL); fibrinogen (0 mg/dL).	Head CT scan: right occipital intracerebral hemorrhage. MR angiogram: bilateral transverse sinus thrombosis extending to right sigmoid sinus & internal jugular vein.Body angio-CT scan (day 3): thrombosis progression towards the posterior half of superior sagittal sinus, bilateral PE & bilateral deep femoral and iliac veins thrombosis reaching infrarenal cava.	IV fluid, empiric antibiotics, hypertonic saline, Fibrinogen, platelet & plasma transfusion; later lopinavir, ritonavir, hydroxychloroquine, & azithromycin; LMWH	Favorable; recovery within 24 days	[78]

↑, increased; ↓, decreased; AF, atrial fibrillation; aPTT, activated partial thromboplastin time; ACA, anterior cerebral artery; ALT, alanine aminotransferase; ARDS, acute respiratory distress syndrome; AST, aspartate aminotransferase; CBC_dif_, complete blood counts with differential; CK, creatine kinase; COVID-19, coronavirus disease 2019; CRP, C-reactive protein; CSF, cerebrospinal fluid; DWI, diffusion-weighted imaging; ESR, erythrocyte sedimentation rate; EVD, external ventricular drainage; F, female; GGT, gamma glutamyl transferase; Hgb, hemoglobulin; ICA, internal carotid artery; IL, interleukin; INR, international normalized ratio; LAC, lupus anticoagulant; LDH, lactate dehydrogenase; LFT, liver function test; LMWH, low molecular weight heparin; M, male; MCA, middle cerebral artery; NIHSS, National Institutes of Health Stroke Scale; NR, not reported; PCA, posterior cerebral artery; PE, pulmonary embolism; PICA, posterior–inferior cerebellar artery; PMN, polymorphonuclear; PT, prothrombin time; RFT, renal function test; SAH, subarachnoid hemorrhage; SARS-CoV-2, severe acute respiratory syndrome coronavirus 2; t-PA, tissue plasminogen activator; WBC, white blood cell; WFD, word finding difficulty.

## Data Availability

Data sharing not applicable.
